# Compressive Multispectral Spectrum Sensing for Spectrum Cartography

**DOI:** 10.3390/s18020387

**Published:** 2018-01-29

**Authors:** Jeison Marín Alfonso, Jose Ignacio Martínez Torre, Henry Arguello Fuentes, Leonardo Betancur Agudelo

**Affiliations:** 1 GIDATI Research Group, Universidad Pontificia Bolivariana, 050031 Medellín, Colombia; jeison.marin@upb.edu.co (J.M.A.); leonardo.betancur@upb.edu.co (L.B.A); 2 GHDwSw Research Group, ETSII, Campus Energía Inteligente, Universidad Rey Juan Carlos, 28933 Madrid, España; 3 HDSP Research Group, Universidad Industrial de Santander, 680002 Bucaramanga, Colombia; henarfu@uis.edu.co

**Keywords:** spectrum cartography, compressive sensing image (CSI), multispectral model

## Abstract

In the process of spectrum sensing applied to wireless communications, it is possible to build interference maps based on acquired power spectral values. This allows the characterization of spectral occupation, which is crucial to take management spectrum decisions. However, the amount of information both in the space and frequency domains that needs to be processed generates an enormous amount of data with high transmission delays and high memory requirements. Meanwhile, compressive sensing is a technique that allows the reconstruction of sparse or compressible signals using fewer samples than those required by the Nyquist criterion. This paper presents a new model that uses compressed multispectral sampling for spectrum sensing. The aim is to reduce the number of data required for the storage and the subsequent construction of power spectral maps with geo-referenced information in different frequency bands. This model is based on architectures that use compressive sensing to analyze multispectral images. The operation of a centralized manager is presented in order to select the power data of different sensors by binary patterns. These sensors are located in different geographical positions. The centralized manager reconstructs a data cube with the transmitted power and frequency of operation of all the sensors based on the samples taken and applying multispectral sensing techniques. The results show that this multispectral data cube can be built with 50% of the samples generated by the devices, and the spectrum cartography information can be stored using only 6.25% of the original data.

## 1. Introduction

Spectrum occupancy information is currently used to make an efficient usage of the spectrum [1]. This information allows central manager to perform spectrum cartography by constructing Interference Maps (IM) with geo-referenced information in different frequency bands.

A central administrator constructs these maps by the information sent by spectral sensors. This construction is based on both the spectral power measured by each sensor and its location. The construction of these maps generally follows 5 stages: Filtering and sampling, interpolation, reduction data, data storage and visualization [2]. In the filtering and sampling stage, energy measurements in different bands are continuously taken at all possible locations by the spectral sensors. In the second stage, different interpolation techniques are used to estimate spectral power values at points that have not been measured. Then, reduction data strategies are used to manage more efficiently the huge amount of recorded data by the spectral sensors. The store stage allows researchers and businesses to analyze aspects as environment modeling for cognitive radio systems, inference models or wide-band spectrum modeling and prediction. This analysis requires big data management and other methods for storing, transmitting and processing the spectral information. The final stage allows operators to visualize the information properly. This stage is used to perform spectrum availability analysis and optimize the network, among other processes.

The construction of IM for spectrum cartography have been extensively studied in the field of spectrum management. In [2] the authors made a survey on the techniques used for the development of interference maps.

One of the first representative works in the construction of IM is [3]. In this work, Ayala et al. constructed IM by locating some spectral sensors in certain geographical points. The sensors send spectral information to a server, and the power levels of all the points in the area are predicted using interpolation algorithms. In [4], the authors presented a spline-based approach to field estimation, where the estimated field enables cartographing the space-frequency distribution of power generated by active RF sources.

In [5,6,7,8], the authors introduced compressive sensing (CS) techniques for the building of IM. In their works, a central manager constructs the maps by CS techniques rather than interpolation techniques, taking advantage of the sparsity in the model of location of the sensors. In  [9], the authors applied CS techniques in spectral signals, taking in count the sparsity of the users occupation in frequency or time domain. However, this work does not focus on the construction of IM.

Currently the construction of IM demands to storage, transmit and process an amount of data that is in the order of thousands of Terabytes [2]. This amount of data requires the development of reduction data strategies that facilitates all the computational processes involved in the construction of these maps. In this sense, several works have used CS techniques as reduction data strategy [5,6,7,8], in the context of cartography. These works have focused only on the spatial sparsity.

In this paper, we consider the possibility to apply a previous subsampling process in the frequency domain. The aim is to reduce the amount of data send by the sensors, which are subsequently processed into the management center. In this way, we can take advantage both spatial and frequency sparsity. Besides, this strategy allows us to analyze simultaneously several IM, since the research works have addressed this problem by processing the IM separately.

We propose a Compressive Sensing Multiespectral Cartography (CSMC) model to construct IM. The proposed model conceives several IM as a data cube of multispectral images [10], which allowed us to apply compressive sensing image (CSI) techniques [11]. In addition, we propose a CSMC architecture for the Spectral Management in a wireless network. This architecture defines the components of the proposed model and the tasks of these components.

Since the data processing in CSI are consistent with the physic model used for acquisition of multispectral images [12], it was required to adapt some stages of the traditional CSI technique and include some new ones. In this sense, our CSMC model takes in consideration aspects as: the spectrum sensing process, the spectrum management devices, operation of radio spectral sensors and smart wireless devices.

We tested our CSMC model with spectral signals acquired by USRP 200 B Software Defined Radios (SDR). Based on these acquired signals, we construct multispectral data cubes up to 96 multispectral maps, which have a size around 6 million samples. We analyze the performance of our model by measuring the PSNR between the original map and the CSMC map. We show how the PSNR is affected when both the number of sensors and IM are increased. The results show that CSMC allows the use of only a 50% of the samples generated by the sensors, as well as, the spectrum cartography information can be stored using only a 6.25% of the original data. The reconstruction process achieved a PSNR above 24 dB for cubes of 96 IM. A higher PSNR can be achieved by using a lower number of maps per data cube.

Recent advances on Urban Sensing [13] and Participatory Sensor Network [14] suggest that in future generation scenarios is feasible to use smart devices as spectrum sensors. In this sense, the proposed model could facilitate the spectrum management in a CSMC network, in the context of smart cities.

The paper is structured in the following sections: Section 1.1 presents the CS theory and how it can be used in multispectral image. Section 1.2 shows the comparison between CSI and CSMC model. Section 2.1 describes the functional architecture of the whole sensing system. In Section 2.2 the CSMC math model is formally presented. The key aspects of proposed model are described in Section 2.3, Section 2.4 and Section 2.5. Section 3 shows the results regarding the implementation of the proposed model. Section 4 shows a discussion of the obtained results. Finally, the conclusions are presented in Section 5.

### 1.1. Compressive Sensing and Compressive Spectral Imaging

Compressive Sensing (CS) is a signal processing technique that can be used for reducing the number of samples in the spectrum sensing operation. CS allows the capturing and reconstruction of a signal using far less samples than those required by traditional approaches [15].

A signal $s\in R^{N}$ is *K*-sparse if ${∥s∥}_{0}=|\left(s\right)|=|s_{\left(k\right)}\neq 0:k=1,\ldots ,N\}|\leq K$, where s has at most *K* non-zeros values. It is possible that the signal has less non-zeros values in another representation basis $\Psi \in R^{N\times N}$ where f=Ψs. CS takes advantage of the sparsity principle of the signals in order to apply sensing protocols that capture the essential information of the signal with a small number of samples. The sensing process can be represented by

(1) g=Φf=ΦΨs,

where $\Phi \in R^{M\times N}$ is a sampling matrix. Note that Equation (1) is an undetermined linear system if M≪N, but if f is sparse, it is possible to find a unique solution solving

(2) $${\hat{f}}_{\ell_{0}}=\arg\;\min_{f}{∥f∥}_{0}\;\mathrm{subject}\;\;\mathrm{to}\;\Phi f=g.$$

CS generally involves solving

(3) $${\hat{f}}_{\ell_{1}}=\arg\;\min_{f}{∥f∥}_{1}\;\mathrm{subject}\;\;\mathrm{to}\;{∥\Phi f-g∥}_{2}\leq \epsilon .$$

or the equivalent convex unconstrained optimization problem:

(4) $$\min_{f}\left(\frac{1}{2}{∥\Phi f-g∥}_{2}^{2}+\lambda {∥f∥}_{1}\right).$$

where ${∥\cdot ∥}_{2}^{2}$ is the Euclidean norm and ${∥\cdot ∥}_{1}$ is the $\ell_{1}$ norm.

Compressive Spectral Imaging (CSI) is an interesting CS application where the data of a multispectral image involves a large amount of spatial and spectral information that can be represented with fewer compressive samples. In some cases, the amount of data in CSI can be reduced up to 90%. In this field, three of the most remarkable CSI architectures are the spatio-spectral encoded compressive HS imager (SSCSI) [16], the coded aperture snapshot spectral imagers (CASSI) [12] and snapshot colored compressive spectral imager (SCCSI) [11].

**Figure 1:**
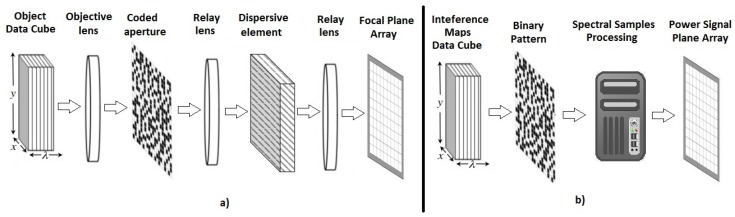
Comparison between architectures. (**a**) coded aperture snapshot spectral imagers (CASSI); (**b**) Compressive Sensing Multiespectral Cartography (CSMC).

In CSI, the multispectral image is modeled as a data cube $f\in R^{M\times N\times L}$ where M×N are the spatial dimensions and *L* is the number of spectral bands. CSI measurements can be modeled as Equation (1) and the signal can be reconstructed by solving the optimization problem Equation (4). In this case, the sampling matrix Φ corresponds to an optical system. For instance, CASSI system [12] is an architecture that attains CSI measurements, in three main steps: first encoding the information with a code aperture pattern, second using a prism as a dispersive element that shifts the spectral information and finally impinging in a focal plane array (FPA) detector. The process is shown in the Figure 1a.

### 1.2. Analogy Between Compressive Spectral Imaging and Compressive Sensing Multispectral Cartography Architectures

Let us consider a scenario where there are *J* sensors located in a geographical area of M×N square meters. The sensors are broadcasting power information of *L* spectral bands and the location of the sensors is known. It is possible to model this power-spectral information as a group of IM in different frequencies by building a 3D data cube. In this 3D data cube, the *X* and *Y* axis correspond to different IM and the *Z* axis corresponds to the different spectral bands. Our proposal is called Compressive Sensing Multispectral Cartography (CSMC) and combines both the CS and CSI techniques to significantly reduce the data involved in the process.

The CSI and CSMC architectures have several key points in common:

- In CSI, the image is modelled as a 3D data cube where each pixel has a (x,y) position and a spectral signature that is represented by the vector $\Phi =[\lambda_{1},\lambda_{2},\cdots ,\lambda_{L}]$. In CSMC, each sensor is located in a (m,n) geographical point and can sense the spectrum in L frequency bands, whose central frequencies are represented by the vector $\lambda =[\lambda_{1},\lambda_{2},\cdots ,\lambda_{L}]$.
- In CSI, each pixel has a colour intensity that depends on its wavelength. In CSMC, each pixel corresponds to a power level, in dB, that depends on the spectral band measured. Therefore, chromatic intensity in CSI corresponds to spectral power level in CSMC.
- CSI is applied to the visible electromagnetic spectrum, while CSMC is applied to the electromagnetic radio-microwaves spectrum.
- CSI uses aperture codes to select what to sample in the CS process. These codes allow the selective passage of the light into the multispectral digital cameras. In the same style, CSMC uses binary patterns to select what spectral power values will be acquired by the sensors, and to select the spectral band.
- In CSI, the spectral imaging information of a 3D cube is projected onto a single 2D plane called Focal Plane Array (FPA). The projection is developed as linear combinations of the coded and spectral dispersed versions of the underlying signal. In CSMC, the spectral manager builds a Power Signal Plane Array (PSPA) as linear combinations of the coded and spectral versions of the power spectral signal.
- In CSI, the original multispectral image is reconstructed only using the FPA samples, solving the optimization problem described. In CSMC, the spectral manager takes the 3D data cube that was built and solves the optimization problem previously described. In both cases, the 3D original data cubes are unknown.

The CSMC measures, as in the CSI case, are processed in three stages: First, the spectral information is sampled using binary patterns, then, the samples are computationally processed. Finally, the information is stored in the PSPA (Figure 1b).

## 2. Materials and Methods

Eskola et al. [2] show the stages of creating Cartographic Interference Maps: Filtering and sampling, Interpolation, Reduction of data, Data Storage and Visualization. Following this approach, our CSMC model implements these stages as follows:

- **Filtering and Sampling** Compressive multidimensional sensing is used both in frequency and spatial domains. The sampling process is developed by binary patterns, which are created by the system. This process will be discussed in Section 2.3.
- **Interpolation** Propagation models are used to calculate the power spectral levels in the geographical points where there are no sensors. This process will be explained later in the experimental implementation (Section 3.3).
- **Reduction of data** We propose three multispectral architectures to reduction of the data. This reduction allows the reduction of sampling and the processing. These architectures will be presented in Section 2.4.
- **Data Storage** For the architectures, the PSPA is represented as a 2D array that is stored in the spectral management’s memory unit.
- **Visualization** The Spectral maps are built based on the PSPA data array by solving the optimization problems presented in Section 1.1.

In order to implement CSMC, we first propose a functional architecture based on the three main devices involved: the sensors, the server and the spectrum broker. Then, we describe the algorithms and the mathematical model associated with each stage.

### 2.1. System Description and Associated Functions

**Figure 2:**
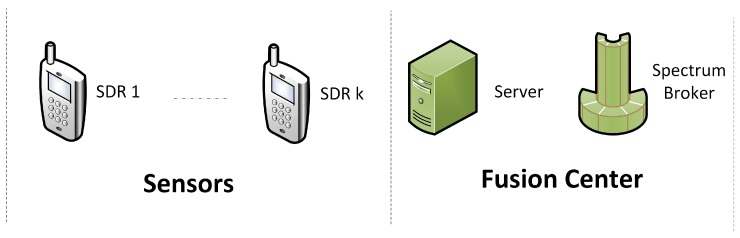
System Components.

The CSMC system is made up of two components: The Fusion Center and the Sensors (Figure 2).

The global target is to make cooperative spectrum sensing, where several sensors send spectral power information to the Fusion Center, which processes the information with its two main components: a Server and a Spectral Manager. The Spectral Manager, commonly called Spectrum Broker (SB), is known in wireless networks as the device that controls and distributes spectral resources [17,18]. In our context, the sensors correspond to SDR, which can modify their own spectrum configuration, modulation scheme or transmission level power, and specially, they can measure the power spectrum values of a given frequency range.

**Figure 3:**
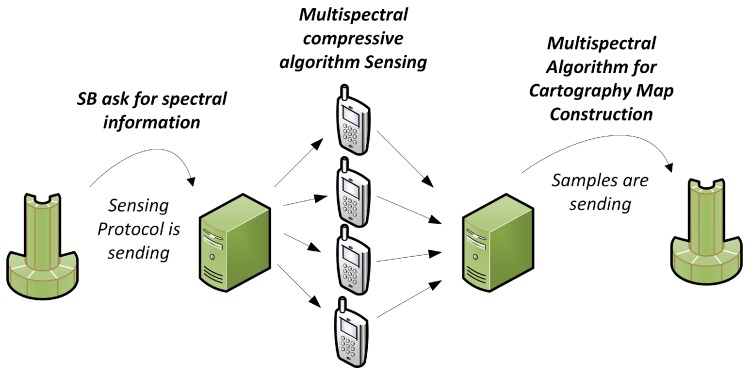
Sensing Process.

The three components of the system are inter-related and have specific functions. These components have inputs and outputs clearly defined. Figure 3 shows the general process associated with the construction of a new interference map, where all the sensors of the network send information to the Fusion Center.

**Figure 4:**
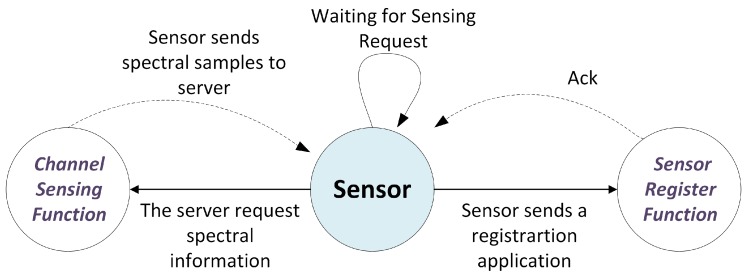
Sensor Functions.

The functions of the sensors are shown in Figure 4 and correspond to channel sensing and sensor registering functions. The Sensor Registering Function is activated when a new sensor log into the system, so therefore the server has an updated list of all the sensors and their geographical location. The Channel Sensing Function is activated when the Fusion Center asks the sensors to send their spectral power values. It is important to emphasize that in this CSMC proposal, the sensors only acquire and send the values requested by the Fusion Center. Therefore, the server must send the specific code that defines which samples must be acquired together with the sensing request.

**Figure 5:**
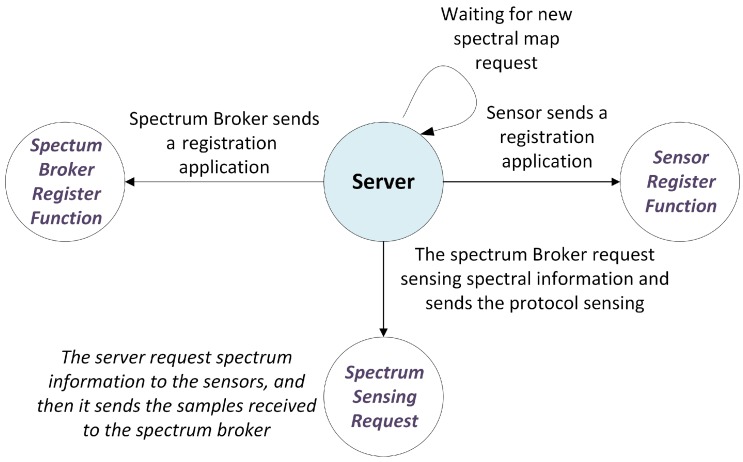
Server Functions.

The functions related to the Server are shown in Figure 5. The Server has two main functions: serving as a bridge between the SDRs and the SB, and registering. There are two main registering functions: the SB register and the register of the sensors. The Server stores the list of sensors and Spectrum Brokers registered in the system. The advantage of being able to work with different Spectrum Brokers is that it allows the system to scale and work with different domains of sensors. For this reason, if there is more than one SB, the sub-system is called Spectrum Broker Domain (SBD).

**Figure 6:**
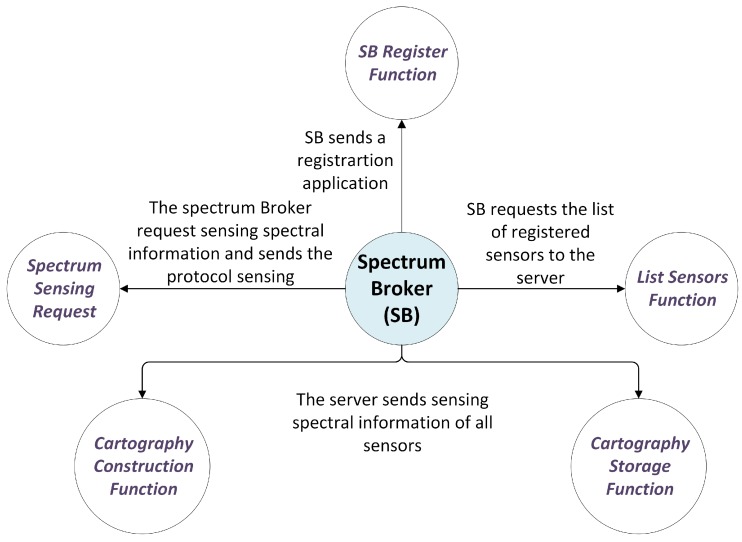
Spectrum Broker Functions.

Finally, the five functions of the SB are shown in Figure 6. The SB register function allows the SB to be registered in the system. The List Sensors function let the Server to request the list of sensors assigned to its domain. In this way, the Server can design the sensing algorithms or the binary patterns, and generates reports. The Spectrum Sensing Request function allows the activation of the sensors, which sense the Spectrum and acquire the power samples. The Cartographic Construction function constructs the cartographic map. The last function, Cartography Storage, allows the system to store the IM.

### 2.2. Compressive Spectrum Sensing Multispectral Model

Let *J* be the number of sensors associated with an interference map. Let $f_{j}\left(t\right)$ the received signal at the *j* sensor, where 1≤j≤J. The signal $f_{j}\left(t\right)$ has the form,

(5) $$f_{j}\left(t\right)=h_{j}\left(t\right)*s_{j}\left(t\right)+n\left(t\right),$$

where $h_{j}\left(t\right)$ is the channel impulse response, $s_{j}\left(t\right)$ is the transmitted signal, n(t) is the white Gaussian noise and ∗ denotes convolution. By performing a Fourier transformation on the received signal, we can obtain

(6) $$f_{j}\left(w\right)=h_{j}\left(w\right)s_{j}\left(w\right)+n\left(w\right),$$

where $f_{j}\left(w\right)$, $h_{j}\left(w\right)$, $s_{j}\left(w\right)$ and n(w) corresponds to the Fourier transformation of $f_{j}\left(t\right)$, $h_{j}\left(t\right)$, $s_{j}\left(t\right)$ and n(t) respectively.

Once the signal is acquired by the SB, it performs a discretization process. Let $f_{j}={\left[f_{j}\left(w_{0}\right)\cdots f_{j}\left(w_{L-1}\right)\right]}^{T}\in C^{L\times 1}$ be a vector with *L* frequency-domain samples, where $w_{l}=l\Delta w$, 0<l<L−1 and Δw=2π/L. The SB can built *L* IM, that is, one interference map for each subchannel $w_{l}$. In this work, each interference map corresponds to a frequency $\lambda_{l}=w_{l}/2\pi$. Since there are *J* wireless smart devices connected, the SB builds an interference map based on the information of all the devices.

Let $\tilde{A}$ be an area of $\tilde{N}\times \tilde{M}$ meters, and let *A* be the discretized version of $\tilde{A}$ with a grid of N×M points. We propose to represent several IM as a N×M×L data cube where N×M corresponds to the spatial location of the power transmitted by wireless devices, and *L* are the possible spectrum slot bands. Let $F_{\lambda }\in R^{N\times M}$ be a interference map at frequency λ. Let $F\in R^{N\times M\times L}$ be a 3D data cube with *L* interference maps, and let $\left\{\lambda_{1},\lambda_{2},\cdots \lambda_{L}\right\}$ be the respective central frequency of each map.

**Figure 7:**
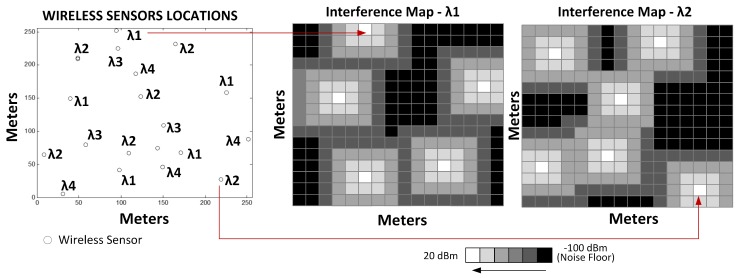
Power handling images in 2 frequency bands.

For example, Figure 7 shows the representation of two IM in an scenario with several wireless devices transmitting at 20 dBm. In this scenario the wireless devices are located in a geographical area *A* and are transmitting in four possible bands: $\lambda_{1}$, $\lambda_{2}$, $\lambda_{3}$ and $\lambda_{4}$, i.e, L=4. The grayscale pixels of each interference map represents power levels, where white is the highest power level and black the lowest one.

**Figure 8:**
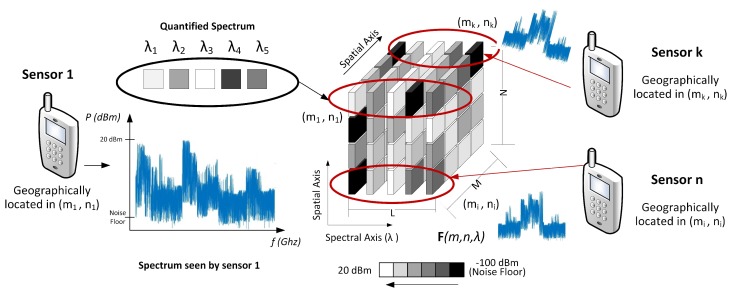
Data cube construction based on the spectral information of the sensors.

In order to construct the 3D data cube F of the proposed model, suppose that there are *J* sensors located in *A*. We are considering that the smart wireless devices can also operate as sensors. Figure 8 shows how the data cube is built using the samples sent from each sensor.

### 2.3. Binary Patterns and Transmittance

The data cube shown in Figure 8 is constructed using all samples of the spectral signals sent by the sensors, and knowing the values of all geographic points of the maps. However, in CSMC model only compressive samples in the frequency and spatial domains are used to construct the IM. In this sense, we propose the use of binary patterns that define which samples to take in each domain.

**Figure 9:**
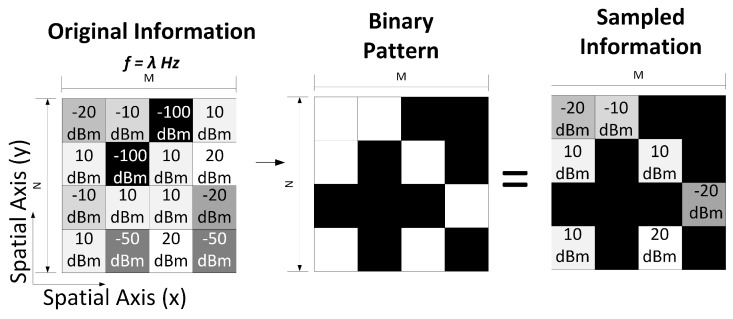
Binary pattern in one spectral band.

Let $T_{\lambda_{i}}\in R^{N\times M}$ be a binary pattern matrix associated with the frequency band $\lambda_{i}$, where its elements are ones or zeros, and 1≤i≤L. Let $T\in R^{N\times M\times L}$ be the 3D matrix with all $T_{\lambda_{i}}$ 2D matrices. Each matrix $T_{\lambda_{i}}$ should select the coordinates (n,m) of $F_{\lambda_{i}}$ that will be sampled by the SB, and which will be rejected. Specifically, we have a matrix $T=\left(t_{n,m,l}\right)\in R^{N\times M\times L},t_{n,m,l}\in \left\{0,1\right\}$ where $t_{n,m,l}=1$ represents a transmissive element and $t_{n,m,l}=0$ represents a block element. Note that if $t_{n,m,l}=1$, $f_{n,m,l}$ is sampled, and if $t_{n,m,l}=0$, $f_{n,m,l}$ is rejected. Figure 9 shows an example of a binary pattern $T_{\lambda }$ of 16 pixels that defines the samples to take in the λ spectral band. Note that the binary pattern discards 9 samples.

**Figure 10:**
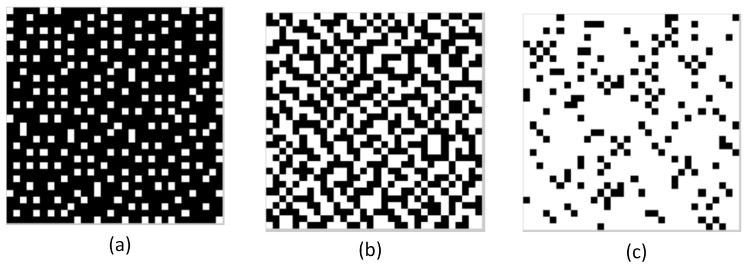
Three binary patterns with different transmittance. (**a**) T=0.2; (**b**) T=0.5; (**c**) T=0.9.

Let $T_{\lambda_{i}}$ be the transmittance value of a binary pattern $T_{\lambda_{i}}$ , where 0≥T≥1. This value depends on the percentage of ones in the $T_{\lambda_{i}}$ 2D matrix. The transmittance value of the $T_{\lambda_{i}}$ is calculated as

(7) $$T_{\lambda_{i}}=\sum_{n=0}^{N-1}\sum_{m=0}^{M-1}\frac{t_{n,m}}{NM},$$

where *N* are the horizontal pixels and *M* the vertical pixels of $T_{\lambda_{i}}$. For example, T=0.2 means that the 20% of the binary pattern are transmissive and the remaining 80% are blocking. Figure 10 shows three binary patterns with different transmittance values. The binary patterns are constructed following the methodology used in [12], where the coded apertures are optimally designed using the restricted isometry property (RIP) to provide the optimization criteria.

### 2.4. CSMC Architectures

In this work, we propose three architectures based on the operation of binary patterns to perform the CSMC. The sampling process is different in each architecture. In architectures one and three, only one binary pattern is designed for all *L* bands. Therefore, in these cases, the same samples and the same geographic points are selected in all IM. In architecture two there are a binary pattern different for each interference map, therefore, different spectral samples and different geographic points are selected, depending on the map that is being processed.

In CSMC architectures, the main objective is to map all IM in a single 2D matrix called Power Signal Plane Array (PSPA). The key aspect of the CSMC model is the subsequent construction of the IM from the PSPA. Let $P\in R^{U\times V}$ be the PSPA matrix, where in architectures one and two, U=N and V=M, and in architecture three U=N and V=M+L−1. The pixels of P matrix are calculated by linear combinations of the voxels of F and T. Specifically the pixels of the matrix P in the three architectures are calculated by

(8) $$\begin{array}{cc} P_{jl}=\sum_{k=0}^{L-1}F_{jlk}T_{jl} & \left(a\right)\\ P_{jl}=\sum_{k=0}^{L-1}F_{jlk}T_{jlk} & \left(b\right)\\ P_{jl}=\sum_{k=0}^{L-1}F_{j(l+k)k}T_{j(l+k)} & \left(c\right) \end{array}$$

where the Equation (8a–c) corresponds to architecture one, two and three respectively.

**Figure 11:**
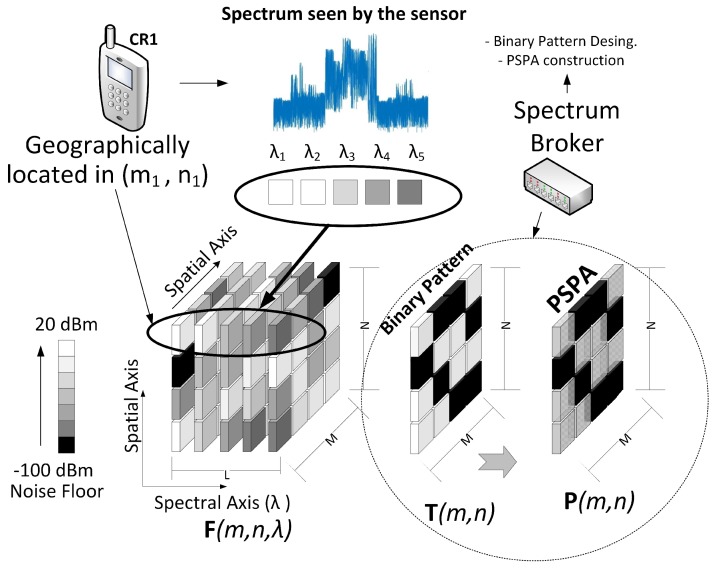
Architecture 1 for CSMC.

**Figure 12:**
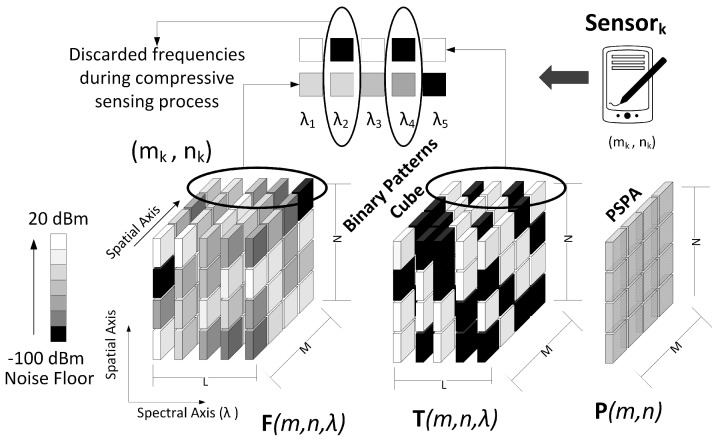
Architecture 2 for CSMC.

**Figure 13:**
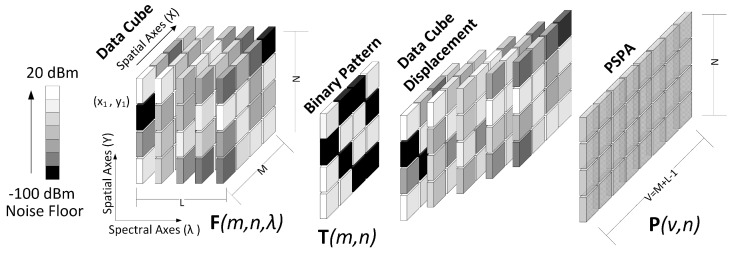
Architecture 3 for CSMC.

The first architecture is shown in Figure 11. In this case, $\{T_{\lambda_{i}}=T_{\lambda_{j}}\;\forall \;1\leq i,j\leq L$}. For this reason, we can represent T as a 2D matrix. In this architecture the power level information of several geographical points in all the bands is discarded, therefore, it is possible to have an ill-conditioning problem. In the second architecture, shown in Figure 12, $\{T_{\lambda_{i}}\neq T_{\lambda_{j}}\;\forall \;1\leq i,j\leq L,\;i\neq j$}. This mean that T is a 3D matrix. Finally, in order to get a closer analogy with the CASSI system, we propose Architecture 3 (Figure 13), where the PSPA is calculated by the linear combination of displaced data samples. In this case also T corresponds to 2D matrix.

### 2.5. The IMs Data Cube Construction

In all the architectures, the PSPA matrix can be represented by a one-dimensional vectorized array. Let $p\in R^{UV\times 1}$ be the vectorized representation of the matrix $P\in R^{U\times V}$, which can be rewritten in the form of an underdetermined system of linear equations by

**Figure 14:**
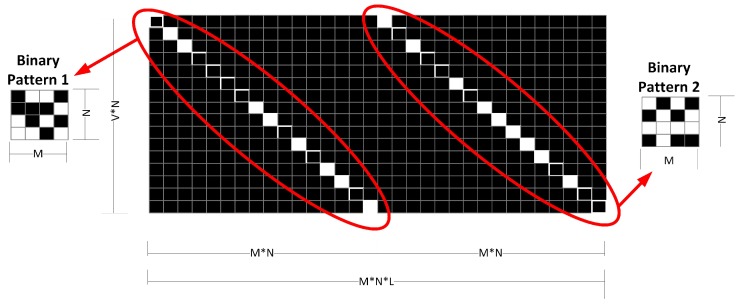
The sensing matrix Φ.

(9) p=Φr=ΦΨf

where $f\in R^{MNL\times 1}$ is the vectorized representation of the IM data cube F. $\Psi \in C^{MNL\times MNL}$ is a representation basis and $\Phi \in R^{VN\times MNL}$ is the multispectral sensing matrix that accounts for the effects of the binary patterns. Note that (9) corresponds to the Equation (1). In multispectral imaging it is common to use the Kronecker representation basis [12]. Figure 14 shows the structure of the sensing matrix Φ in the case where L=2, and the second architecture is used (V=M). The diagonal patterns of Φ correspond to the binary patterns $T_{\lambda_{1}}$ and $T_{\lambda_{2}}$. The structure of Φ in the architecture one is similar, except that $T_{\lambda_{1}}$ would be equal to $T_{\lambda_{2}}$. In architecture three V=M+L−1, and the second set of diagonal pattern would be displaced.

Several numerical algorithms can be used to solve the inverse problem in Equation (3) [19]. We select the Constrained-Split Augmented Lagrangian Shrinkage algorithm: C-SALSA [20] to construct the IM data cube from compressed measurements. We select C-SALSA because our IM images are characterized by spatial smoothing and the data cube present high spectral correlation. In this case, where total variation (TV) base regularization can be used, C-SALSA was found to be faster than other state-of-the-art methods [20], therefore, this algorithm is widely used in recent works [21,22]. C-SALSA solve the optimization problem

(10) $$\begin{array}{c} \min_{r,w\in R^{N},v\in R^{M}}{∥w∥}_{1}+\iota_{E(\epsilon ,I,0)}\left(v\right)\\ \mathrm{subject}\;\;\mathrm{to}\;w=\Psi f\;v=\Phi r-P, \end{array}$$

where E(ϵ,Φ,P) is an ellipsoid that corresponds to the feasible set in problem Equation (3),

(11) $$E\left(\epsilon ,\Phi ,P\right)=\{r\in R^{N}:∥\Phi r-P{∥}_{2}\leq \epsilon \}$$

In Equation (10), E(ϵ,I,0) is a closed ϵ radius Euclidean ball centered on the origen of $R^{D}$, and $\iota_{S}:R^{D}\to \bar{R}$ denotes the indicator function of set $S\subset R^{D}$,

(12) $$\iota_{S}\left(s\right)=\left\{\begin{array}{c} 0\\ +\infty \end{array}\right.\left.\begin{array}{c} \;\mathrm{if}\;s\in S\\ \;\mathrm{if}\;s\notin S \end{array}\right.$$

The SB performs the data cube construction process described in the Algorithm 1.

**Algorithm 1** The general process for building the data cube with the IM**Require:** There are *J* sensors in the area. There are *L* spectral bands. There are M×N geographic points.1:The **SB** requires *L* IMs (one for each spectral band).2:**for** k←1,S **do**3:  The **SB** defines the samples to request from the ${\mathrm{Sensor}}_{k}$ using a binary pattern.4:  The **SB** request the samples from the ${\mathrm{Sensor}}_{k}$5:**end for**6:The **SB** builds the model of the Equation (9).7:The **SB** solves the optimization problem of the Equation (10).


## 3. Results

### 3.1. Preliminary Simulations

A first set of simulations was set to prove the correct behavior of every architecture in the construction and store phases of the IM. Additionally, these simulations let us study how the change of the transmittance value impact the architectures. This analysis involved 3D multispectral cubes with 8 bands (8 cartographic maps) in a geographic area of 256×256 cells. The cubes were sampled and the architectures tested using binary patterns of transmittance values from 0.1 to 0.9.

**Figure 15:**
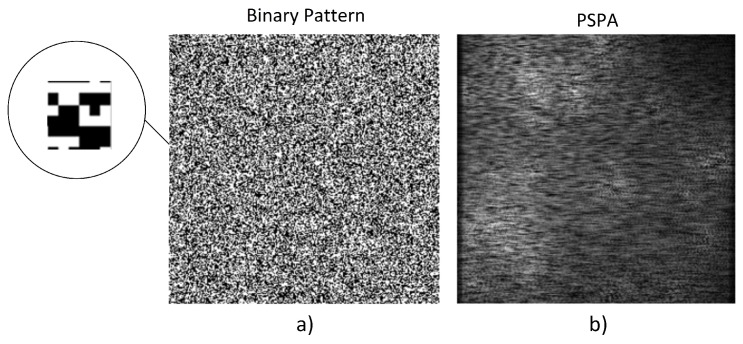
(**a**) Binary pattern used in the simulations; (**b**) Power Signal Plane Array (PSPA) calculated in the simulation.

All the proposed architectures were initially implemented with the 0.5 transmittance binary pattern shown in Figure 15a. The simulation runs on a geographical area of 256×256 cells with 30 SDR transmitting at 20 dBm distributed in all 8 possible bands. Figure 15b shows the PSPA calculated.

**Figure 16:**
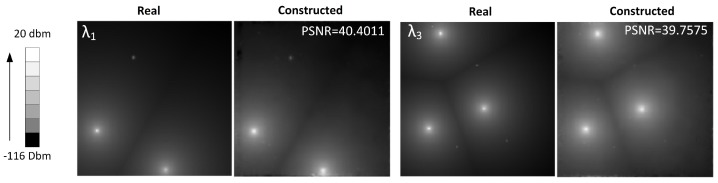
Interference Maps (IM) Constructed by the Spectrum Broker (SB).

Figure 16 compares the ideal interference map with the interference map built for the spectral bands $\lambda_{1}$ and $\lambda_{3}$. The interference map was built using the PSPA P matrix and solving the optimization problem of Equation (10).

**Figure 17:**
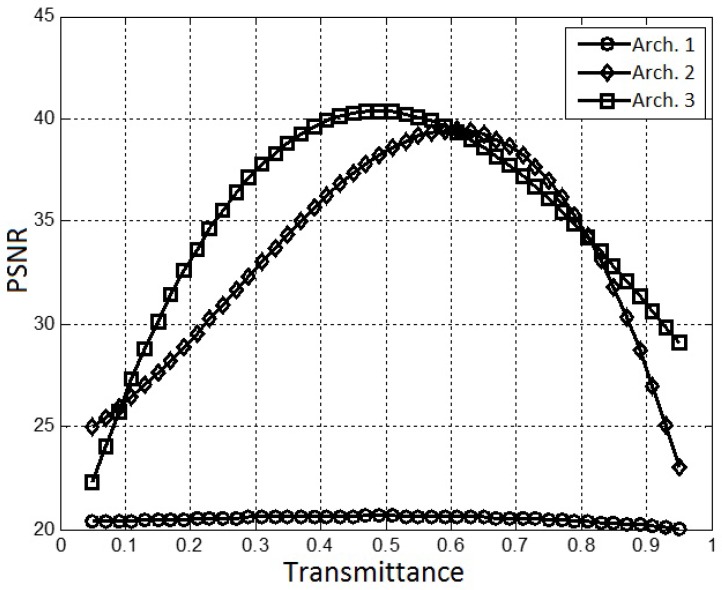
Transmittance analysis of the three architectures.

In order to compare the influence of the binary patterns transmittance values in the different architectures, we carried out a whole set of simulations. Figure 17 shows these results in terms of the Peak Signal to Noise Ratio (PSNR) for the ideal and constructed data cubes.

### 3.2. Decimated Rate Results of Preliminary Simulations

Using the Equation (8a–c), all information of the data cube were condensed in the PSPA. We calculate a decimated rate (DR) by

(13) $$DR=\frac{DN}{NS},$$

where DN corresponds to the total number of original data and NS is the size of the PSPA. Table 1 shows the results for 8 spectral bands using Architecture 2 of the CSMC model. These results are compared with the sampling rate used in previous works with spatial interpolation [3], and with compressive sensing to construct the cartographic maps using the “Orthogonal Matching Pursuit” (OMP) algorithm [5].

### 3.3. Simulations with Experimental Signals

**Figure 18:**
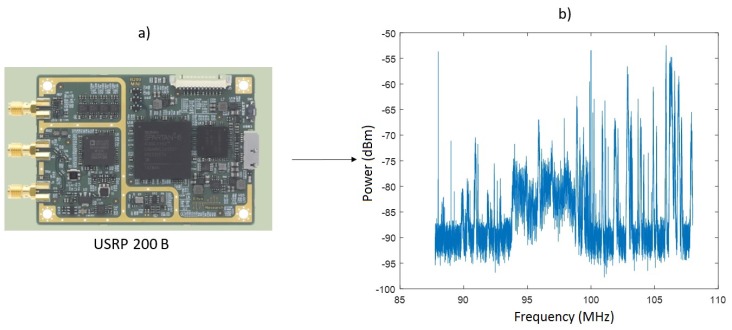
USRP B200-mini Radio.

After analyzing the results of the first simulations, Architecture 2 was selected for extra simulations to test the entire system for different number of bands with real spectral power signals acquired by a SDR *USRP 200B Ettus* with a transmittance value of 0.5. Figure 18 shows the SDR model and an example of the power spectral signal.

**Figure 19:**
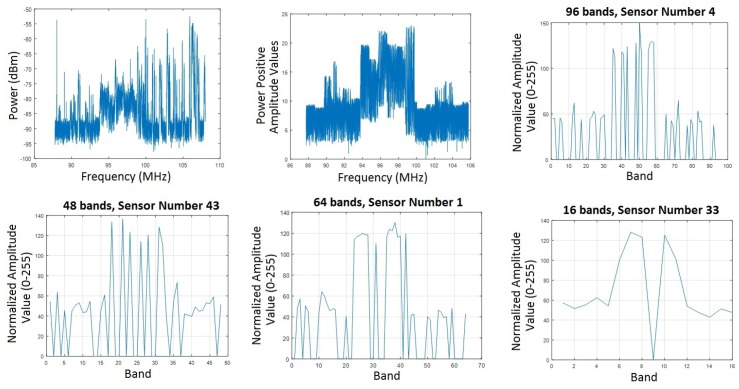
Experimental Signal, Filtering Signal and Quantification in Only 8k bands signals.

The experimental signal has 18.000 power samples in dBm in the frequency domain. The signal was reduced to *L* frequency bands through a filter, where L=8k and 1≤k≤12. Figure 19 shows how the process goes from the experimental signal to the final signal reduced to L=96,64,48 and 16 frequency bands.

In order to compute *L* IM using this signal, we perform simulations where *S* sensors are randomly located in a geographical area of 256×256 cells, where S=10,20,50,100,150 and 200.

**Figure 20:**
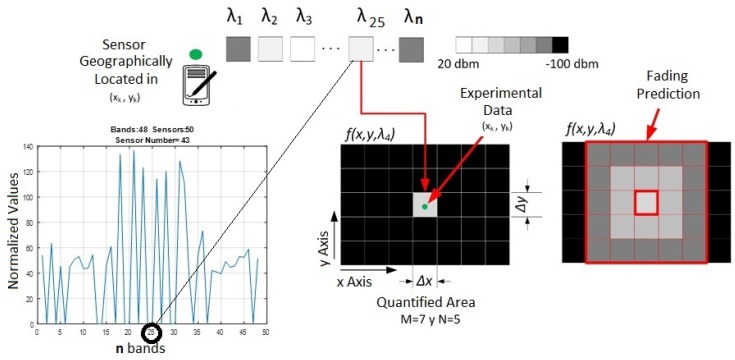
Interference Maps Construction, based on measurements and propagation models.

Each sensor has an assigned spectral signal with the same probability distribution as the experimental signal. In addition, given that the sensors are SDR, each of the sensors have an assigned transmission power in one of the *L* bands; taking this into account, it is possible to calculate the power levels in other geographical points using propagation models (see Figure 20).

**Figure 21:**
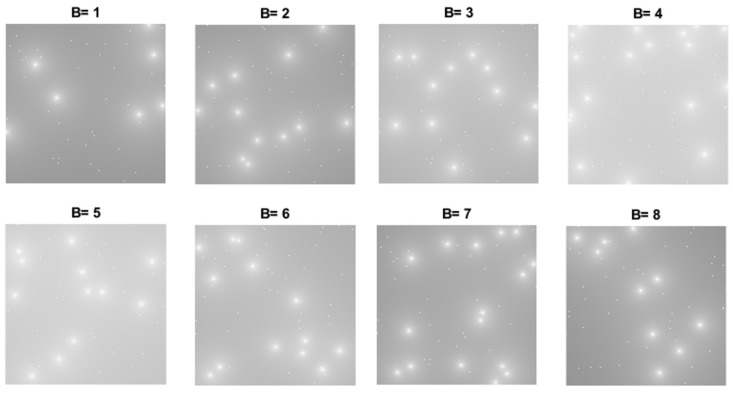
Interference Maps of 8 Bands Mutispectral Data Cube.

Once the *L* IM are built, the multispectral cubes are arranged. Note that altogether there are 6×12=72 data cubes, each with *L* bands and *S* sensors. Figure 21 shows an instance of a data cube with 8 bands, 256×256×8 geographical values and 50 sensors. The sensors in the maps that appears as white points are those that are not broadcasting in that band, so the point is equivalent to a single measurement in that geographical place.

With the data cubes already arranged, the CSMC Architecture 2 was implemented with a 0.5 transmittance value. The PSPA was calculated for each data cube using Equation (8b) and, finally, the data cube with L spectral bands was built. The metrics used to evaluate the quality of the solution were the PSNR for the IM and the Mean Squared Error (MSE) for the spectral signals of the sensors. Finally, we use a Decimated Rate (DR) as a metric for quantifying the data saving; this metric considers the discarded data during the compressive sensing of the signals plus the data compression obtained during the PSPA construction.

**Figure 22:**
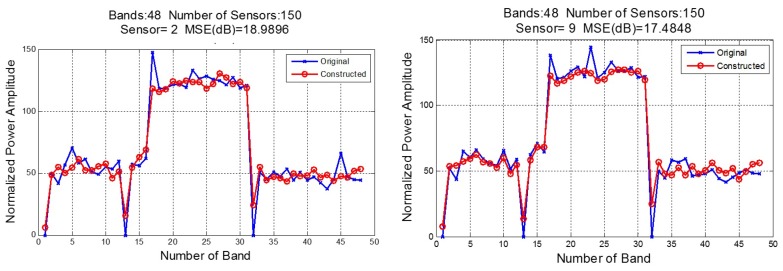
Sensor Spectral Signal Constructed.

Figure 22 shows two examples of the comparison of the MSE for the original power spectral signal (with *L* frequency points) with the reconstructed one. In this case, we compare sensors 2 and 9 in a data cube with 150 sensors and 48 bands.

**Figure 23:**
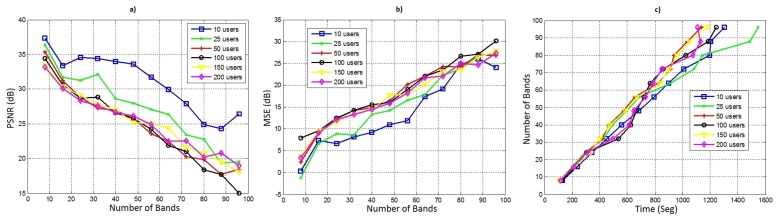
For different number of bands and sensors: (**a**) PSNR between original and constructed maps; (**b**) MSE between original and constructed spectrum signals; (**c**) Time maps construction.

As a global result of the test of the all cubes generated with different number of bands and sensors, Figure 23a shows the result of the mean PSNR for all the original and constructed IM, Figure 23b the mean MSE for all the original and constructed power spectral signals of the sensors and, finally Figure 23c shows the processing time spent in the maps construction for each case.

Table 2 depicts the **DR** calculated for different data cubes with different number of bands.

## 4. Discussion

In the preliminary test of the model, we calculate the PSNR between the real and constructed IM data cubes for all bands. We obtained PSNR values above of 39 dBs, and no artifacts were observed in the reconstructed images.

The data cube construction only involves 50% of the samples of the real data cube, i.e., the binary pattern transmittance is 0.5. In this way, only 50% of the spectral information is sent from the sensors and the SB only calculates 50% of the power levels in the geographical positions.

A considerable reduction is obtained, when the compressive samples are condensed in the PSPA. For example, Table 1 shows a DR value of 16, which corresponds to a 6.25% of the original amount of data of the 3D cube with 8 bands.

We found that there is a value of transmittance that offers the best data cube construction for each architecture. Our results suggest a transmittance between 0.4 and 0.6 offers the better results, i.e., an increment in the transmittance does not necessarily mean an increment in the performance.

It is important to note that the results shown in Figure 17 are consistent with the the relationship between the transmittance of the coded apertures and the RIP determined in [23] for CASSI systems. In [23] the authors find a mathematical expression for the RIP in CASSI, and determine the transmittance that provides the best value of a constant present in these mathematical expression. They found that the best value for the transmittance is 0.5. Therefore, due the great similarity of our CSMC model with the CASSI model, we can affirm that a value of 0.5 for transmittance will generally be a good choice.

Architecture 1 offers the worst performance because all samples of the bands in specific geographical points are discarded.

Preliminary results allowed us to select the Architecture 2 to test the model under different number of bands and sensors. Unlike Architecture 1, note that Architecture 2 has one binary pattern for each band, which allowed us to select frequency samples for all the sensors in different frequency points of the spectrum.

The original data cube, shown in Figure 21, has 8 bands with 256×256×8 geographical points, that corresponds to 524.288 samples. Table 1 shows how the number of data samples required to construct the data cube is greatly reduced. Note that only 32.768 samples were required to construct the 524.288 samples data cube, which corresponds to RD=16 for 8 bands.

In our model, the data reduction is constant whatever the number of bands, therefore the DR increases with the number of bands (Table 2). However, the bigger the number of bands in the data cube, the lower the quality of the constructed map.

There is a trade-off between decimated rate and construction quality that should be taken into account. The required quality depends on the application. For example, in cases where the spectral signals are used only for establishing whether the channel is busy or free, the reconstruction does not have to be so accurate, in this case, it is only necessary to know if the signal exceeds a fixed threshold. For instance, in Figure 22, despite the MSE value being around 18 dB, the constructed signal takes the form of the original signal and it is possible to establish an occupation channel criterion.

## 5. Conclusions

This paper presents a new model of Compressive Sensing Multispectral Cartography, which is based on Compressive Spectral Imaging Techniques. In the proposed model, a Power Spectral Plane Array (PSPA) is built using the compressive samples of the power spectral information sent by the spectral sensors. The PSPA is modeled as an underdetermined system of linear equations. Finally, from the PSPA, the Spectrum Broker constructs the 3D data cube with the IM, solving a sparse reconstruction problem. The results show that this multispectral data cube can be built with 50% of the samples generated by the devices, and the spectrum cartography information can be stored using up to 6.25% of the original data. We consider that the proposed model is plausible for a near future scenario. Our model could facilitate the spectrum management in a CSMC network in the context of smart cities.

## Figures and Tables

**Table 1:** Decimated rate and mean error in the construction of interference maps for three models—8 spectral bands.

	DN	NS	DR	Error
CSMC model	524.288	32.768	16	24 dBm
MC-OMP model	5.000	400	12.5	2 dBm
Spatial Interpolation model	80.000	20.000	4	7 dBm

**Table 2:** Decimated rate calculated for different data cubes.

Number of Bands	DN	NS	DR	PSNR max/min (Maps)	MSE max/min (Sensors Signals)
8	524.288	32.768	16	37.34/33.15 dB	0.35/3.39 dB
16	1’048.576	32.768	32	33.37/30.01 dB	7.28/9.09 dB
24	1’572.864	32.768	48	34.53/28.31 dB	6.65/12.09 dB
32	2’097.152	32.768	64	34.41/27.60 dB	8.15/13.26 dB
40	2’621.440	32.768	80	33.97/26.67 dB	9.22/14.5 dB
48	3’145.728	32.768	96	33.59/26.15 dB	10.95/15.82 dB
56	3’670.016	32.768	112	31.72/24.68 dB	11.88/18.26 dB
64	4’194.304	32.768	128	29.94/22.48 dB	17.39/21.71 dB
72	4’718.592	32.768	144	27.88/22.53 dB	19.20/22.07 dB
80	5’242.880	32.768	160	24.87/20.21 dB	24.72/25.04 dB
88	5’767.168	32.768	176	24.31/20.78 dB	26.07/24.69 dB
96	6’291.456	32.768	192	26.40/18.96 dB	24.10/27.31 dB

## Abbreviations

The following abbreviations are used in this manuscript:

CASSI	Coded Aperture Snapshot Spectral Imagers
CS	Compressive Sensing
CSI	Compressive Spectral Imaging
CSMC	Compressive Sensing Multispectral Cartography
DR	Decimated Rate
FPA	Focal Plane Array
IM	Interference Maps
MSE	Mean Squared Error
PSNR	Peak Signal to Noise Ratio
PSPA	Power Spectral Plane Array
SB	Spectrum Broker
SBD	Spectrum Broker Domain
SCCSI	Colored Compressive Spectral Imager
SDR	Software Define Radio
SSCSI	Spatio Spectral Encoded Compressive Imager
